# Neutrophil to high-density lipoprotein ratio has a superior prognostic value in elderly patients with acute myocardial infarction: a comparison study

**DOI:** 10.1186/s12944-020-01238-2

**Published:** 2020-04-04

**Authors:** Jia-Bao Huang, Yu-Si Chen, Hong-Yan Ji, Wei-Ming Xie, Jie Jiang, Lu-Sen Ran, Cun-Tai Zhang, Xiao-Qing Quan

**Affiliations:** 1 Department of Geriatrics, Tongji Hospital, Tongji Medical College, Huazhong University of Science and Technology, Wuhan, China; 2 Second Clinical School, Tongji Hospital, Tongji Medical College, Huazhong University of Science and Technology, Wuhan, China; 3 Department of General Practice, Shenzhen Longhua District Central Hospital, Shenzhen, China

**Keywords:** Acute myocardial infarction, Neutrophil, High-density lipoprotein cholesterol, Mortality, Recurrent myocardial infarction

## Abstract

**Background:**

The importance of the lipid-related biomarkers has been implicated in the pathological process and prognosis of acute myocardial infarction (AMI). Our work was conducted to discuss and compare the predictive ability of the neutrophil to high-density lipoprotein cholesterol (HDL-C) ratio (NHR) with other existing prognostic indices, for instance, the monocyte to HDL-C ratio (MHR) and the low-density lipoprotein cholesterol (LDL-C) to HDL-C ratio (LDL-C/HDL-C) in elderly patients with AMI.

**Methods:**

Our population was 528 consecutive elderly AMI patients (65–85 years) who were enrolled from Tongji Hospital and grouped according to the cutoff points which were depicted by the receiver operating characteristic (ROC). The Kaplan-Meier curves were plotted with the survival data from the follow-up to investigate the difference between cutoff point-determined groups. Moreover, we assessed the impact of NHR, MHR, LDL-C/HDL-C on the long-term mortality and recurrent myocardial infarction (RMI) with Cox proportional hazard models.

**Results:**

Mean duration of follow-up was 673.85 ± 14.32 days (median 679.50 days). According to ROC curve analysis, NHR ≥ 5.74, MHR ≥ 0.67, LDL-C/HDL-C ≥ 3.57 were regarded as high-risk groups. Kaplan-Meier analysis resulted that the high-NHR, high-MHR and high-LDL-C/HDL-C groups presented higher mortality and RMI rate than the corresponding low-risk groups in predicting the long-term clinical outcomes (log-rank test: all *P* < 0.050). In multivariate analysis, compared with MHR and LDL-C/HDL-C, only NHR was still recognized as a latent predictor for long-term mortality (harzard ratio [HR]: 1.96, 95% confidence interval [CI]: 1.02 to 3.75, *P* = 0.044) and long-term RMI (HR: 2.23, 95% CI: 1.04 to 4.79, *P* = 0.040). Furthermore, the positive correlation between NHR and Gensini score (*r* = 0.15, *P* < 0.001) indicated that NHR was relevant to the severity of coronary artery to some extent.

**Conclusions:**

NHR, a novel laboratory marker, might be a predictor of the long-term clinical outcomes of elderly patients with AMI, which was superior to MHR and LDL-C/HDL-C.

## Introduction

Acute myocardial infarction (AMI), a serious form of coronary artery disease (CAD), is a significant cause of death worldwide, particularly in the elderly population [1]. Actually, the elderly AMI patients who may have poor prognosis could be greatly improved by timely and appropriate intervention measures [2]. Based on this, the discovery of prognosis-related biomarkers could benefit considerably to the oriented treatment and the prognostic evaluation in coronary infarction [3–5]. Thus, to find an efficient predictor is urgently needed.

Nowadays, the general public have more in-depth understanding of the correlation between serum lipid level and CAD [6]. In addition, as for single lipids, some lipid ratios could be well applied to predict CAD and the associations were of comparable magnitude [7]. For instance, the ratio of monocyte to high-density lipoprotein cholesterol (HDL-C) (MHR) [8, 9] and the ratio of low-density lipoprotein cholesterol (LDL-C) to HDL-C (LDL-C/HDL-C) were found to have correlation with poor prognosis in cardiovascular diseases [10, 11].

It was showed that the inflammation and the abnormal lipid metabolism took a critical part in plaque formation and atherosclerosis in the development of AMI [12–15]. Circulating neutrophils played a causative role in the immuno-inflammatory responses of the pathogenesis of atherosclerosis [16]. Neutrophils abundance was strongly connected with the incidence of cardiac adverse events [17]. On the contrary, recent investigations suggested that the HDL-C was inversely related to the risk of CAD and could be highly predictive of its prognosis [18]. What’s more, previous clinical and experimental studies indicated that HDL could regulate the function of activated neutrophils [19]. In contrast, activated neutrophils could also affect the composition and function of HDL [20]. Therefore, we hypothesized that the neutrophil to HDL-C ratio (NHR), a potential new lipid biomarker, might reflect the inflammation level and the lipid profile in a quantitative manner. With these considerations, our work was conducted to compare the predictive value of NHR with other two hematological parameters (including MHR and LDL-C/HDL-C) in elderly AMI patients.

## Methods

### Patients

In current work, the data of 528 patients (65–85 years) with AMI in the Tongji Hospital, Tongji Medical College, Huazhong University of Science and Technology between January 2016 and December 2017 were collected. Permission to accomplish the study was received from the institutional board review committee (TJ-C20141112). This study followed the principles of the Declaration Helsinki.

In our study, the patients were admitted with AMI within 1 month of symptom onset. The eligible population corresponding to AMI diagnosis standards mainly included the patients who was defined as ST-segment elevation myocardial infarction (STEMI, typical symptoms of myocardial ischemia lasting for > 30 min, with ST-segment elevation > 1 mm in ≥2 contiguous leads and/or new onset of left bundle branch block) [21] and without ST-segment elevation myocardial infarction (NSTEMI, a rise of myocardial injury markers in combination with typical symptoms of myocardial ischemia and without ST-segment elevation) [22].

Here were the exclusion criteria. (1) Patients who were younger than 65 or older than 85 years. (2) Patients who had sepsis or trauma. (3) Patients who were diagnosed as active cancer, hematological proliferative diseases, autoimmune diseases, pulmonary arterial hypertension, end-stage liver disease or renal failure. (4) Patients who took medications including any steroid therapy or chemotherapy around the diagnosis period, thrombolytic therapy and glycoprotein IIb/IIIa inhibitors.

### Clinical data collection

Current study evaluated the demographic data from medical records including age, sex, smoking status, and the anamnesis (such as the history of CAD, hypertension, diabetes and cerebrovascular diseases). We collected the vital signs (heart rate, systolic blood pressure, diastolic blood pressure) and Killip class at admission. Besides, the data of pharmacotherapy during hospitalization were included as well.

In all the patients, a routine blood test was performed immediately after admission. Laboratory results included complete blood cell counts (white blood count [WBC] and its subtypes, hemoglobin), aminopherase, creatinine, serum lipid (triglycerides, total cholesterol, LDL-C, HDL-C), N-terminal prohormone of brain natriuretic peptide, and myocardial injury markers. Also, we gathered the result of left ventricular ejection fraction (LVEF) from cardiac ultrasonography.

### Coronary angiography (CAG)

The CAG was performed based on the actual clinical situation and the results were determined by two professional cardiovascular physicians. Based on the clinical findings, physicians would select different treatment strategies (such as percutaneous coronary intervention [PCI], coronary artery bypass grafting [CABG], and medical therapy) in the light of corresponding practice guidelines [23]. Furthermore, we also collected data of thrombolysis in myocardial infarction trial (TIMI) blood flow grade and Gensini score (an angiographic scoring system assessed the severity of a coronary artery) from the electronic medical records database.

### Clinical follow-up and study end points

The endpoints of present study were all-cause mortality and recurrent myocardial infarction (RMI) that happened during the follow-up period. All-cause mortality was defined as death from any cause. RMI was defined as recurrence of AMI whether the reinfarction area located in the original or other site [24]. Follow-up of all patients were began from the first day after discharge until December 2018. The access to information during follow-up was performed through standard telephone interview with patients or their relatives at regular intervals.

### Statistical analysis

NHR, MHR and LDL-C/HDL-C were calculated by the formula respectively: NHR = neutrophil / HDL, MHR = monocyte / HDL and LDL-C/HDL-C = LDL-C / HDL-C. In baseline characteristics analysis, continuous variables were presented as mean ± standard error and tested for normal distribution by the Kolmogorov-Smirnov test. For the comparison of variables between groups, the parametric/nonparametric tests (continuous variables) or Chi-square test (categorical variables) was selected according to the type of variables. Using Receiver operating characteristic (ROC) curves to find out the optimal cutoff points of NHR, MHR and LDL-C/HDL-C. Kaplan-Meier curve for survival analysis was plotted to assess the prognosis between index-determined subgroups with log-rank test. The Spearman correlation coefficient was computed to calculate the correlation between two variables. Univariate analyses were performed to determine the significance of prognostic variables. Any variables examined in the univariate analysis for which the *P* < 0.10 or several conventional risk factors for AMI (such as age, gender, smoking status, and previous history of CAD, hypertension, diabetes) were contained in the forward stepwise multivariate model. A value of *P* < 0.05 was supposed to be statistically significant. All statistical analyses were performed using SPSS Version 22.0.

## Results

### ROC curve analysis

**Fig. 1:**
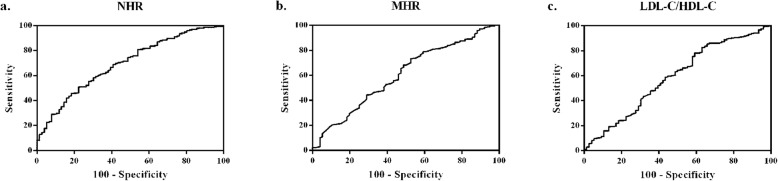
ROC curve analysis for NHR, MHR and LDL-C/HDL-C (**a, b, c**). **a** The AUC for NHR was 0.69 (*P* < 0.001), and the cutoff point was 5.74, with a sensitivity of 77.60% and a specificity of 50.80%. **b** The AUC for MHR was 0.60 (*P* = 0.004), and the cutoff point was 0.67, with a sensitivity of 47.40% and a specificity of 73.40%. **c** The AUC for LDL-C/HDL-C was 0.59 (*P* = 0.017), and the cutoff point was 3.57, with a sensitivity of 34.20% and a specificity of 85.30%. ROC, receiver operating characteristic. AUC, area under the curve. NHR, neutrophil to high-density lipoprotein cholesterol ratio. MHR, monocyte to high-density lipoprotein cholesterol ratio. LDL-C/HDL-C, low-density lipoprotein cholesterol to high-density lipoprotein cholesterol ratio

ROC curve was analyzed in search of the cutoff value of NHR, MHR and LDL-C/HDL-C for the evaluation of long-term clinical outcomes in elderly AMI patients. All of these results were presented in Fig. 1. For NHR, the area under the curve (AUC) was 0.69 (*P* < 0.001, 95% confidence interval [CI]: 0.63 to 0.76). The cutoff point was observed at 5.74, with a sensitivity of 77.60% and a specificity of 50.80%. (Fig. 1a). Accordingly, the AUC for MHR was calculated as 0.60 (*P* = 0.004, 95% CI: 0.53 to 0.68; Fig. 1b). The cutoff value for MHR was found to be 0.67, with a sensitivity of 47.40% and a specificity of 73.40%. Moreover, the AUC for LDL-C/HDL-C was 0.59 (*P* = 0.017, 95% CI: 0.51 to 0.66) with a cutoff point at 3.57 (sensitivity 34.20%, specificity 85.30%; Fig. 1c). Consequently, the AUC for NHR was larger than those for MHR and LDL-C/HDL-C and it preliminarily presented better predictive value for the prognosis in elderly AMI patients.

### Baseline characteristics

As shown in Table 1, the total number of 528 patients were collected and grouped by cutoff points as described above. 333 of the 528 patients were male (63.07%) and the population were aged between 65 and 85 years old (72.66 ± 0.23). Diagnoses included: 268 patients with STEMI and 260 patients with NSTEMI.

**Table 1:** Baseline characteristics of elderly AMI patients

Variable	NHR < 5.74	NHR ≥ 5.74	*P*	MHR < 0.67	MHR ≥ 0.67	*P*	LDL-C/HDL-C < 3.57	LDL-C/HDL-C ≥ 3.57	*P*
	*n* = 249	*n* = 279		*n* = 367	*n* = 161		*n* = 439	*n* = 89	
Age, year	72.54 ± 0.34	72.76 ± 0.32	0.555	72.35 ± 0.27	73.36 ± 0.42	0.048	72.60 ± 0.25	72.93 ± 0.56	0.754
Gender (male), n (%)	150 (60.24%)	183 (65.59%)	0.203	206 (56.13%)	127 (78.89%)	< 0.001	271 (61.73%)	62 (69.66%)	0.157
Smoking status, n (%)	92 (36.95%)	118 (42.29%)	0.210	134 (36.51%)	76 (47.20%)	0.021	167 (38.04%)	43 (48.31%)	0.071
Hypertension, n (%)	144 (57.83%)	169 (60.57%)	0.522	215 (58.58%)	98 (60.87%)	0.623	267 (60.82%)	46 (51.69%)	0.110
Prior CAD, n (%)	40 (16.06%)	30 (10.75%)	0.072	50 (13.62%)	20 (12.42%)	0.708	60 (13.67%)	10 (11.24%)	0.537
Diabetes, n (%)	51 (20.48%)	86 (30.82%)	0.007	94 (25.61%)	43 (26.71%)	0.792	112 (25.51%)	25 (28.09%)	0.613
CVD, n (%)	33 (13.25%)	39 (13.98%)	0.808	51 (13.90%)	21 (13.04%)	0.793	59 (13.44%)	13 (14.61%)	0.770
SBP on admission (mmHg)	132.81 ± 1.50	128.10 ± 1.49	0.013	131.50 ± 1.31	127.65 ± 1.77	0.073	130.14 ± 1.15	131.25 ± 2.75	0.854
DBP on admission (mmHg)	75.65 ± 0.84	75.61 ± 0.94	0.927	76.31 ± 0.76	74.07 ± 1.14	0.042	75.69 ± 0.69	75.34 ± 1.63	0.733
HR on admission (beats/min)	74.56 ± 0.93	80.35 ± 0.96	< 0.001	76.44 ± 0.80	80.31 ± 1.29	0.023	77.11 ± 0.74	80.13 ± 1.78	0.364
Hospitalization day	7.56 ± 0.36	8.99 ± 0.45	0.013	7.99 ± 0.31	9.06 ± 0.66	0.466	8.08 ± 0.31	9.47 ± 0.85	0.290
Diagnosis, n (%)									
STEMI	98 (39.36%)	170 (60.93%)	< 0.001	181 (49.32%)	87 (54.04%)	0.318	216 (49.20%)	52 (58.43%)	0.112
NSTEMI	151 (60.64%)	109 (39.07%)	< 0.001	186 (50.68%)	74 (45.96%)	0.318	223 (50.80%)	37 (41.57%)	0.112
Killip class, III-IV, n (%)	41 (16.47%)	60 (21.51%)	0.142	61 (16.62%)	40 (24.84%)	0.027	81 (18.45%)	20 (22.47%)	0.379
LVEF, %	53.72 ± 0.78	50.45 ± 0.75	0.001	52.85 ± 0.61	50.02 ± 1.11	0.042	51.93 ± 0.61	52.27 ± 1.23	0.464
Creatinine (μmol/L)	84.54 ± 1.70	95.13 ± 2.13	< 0.001	87.59 ± 1.71	95.94 ± 2.34	< 0.001	88.89 ± 1.47	96.29 ± 4.01	0.169
AST (u/L)	56.61 ± 4.44	119.89 ± 7.63	< 0.001	92.17 ± 6.09	85.22 ± 7.04	0.373	89.69 ± 4.97	91.84 ± 13.88	0.584
ALT (u/L)	29.47 ± 1.72	43.56 ± 2.76	< 0.001	34.27 ± 1.72	42.94 ± 3.91	0.004	36.85 ± 1.94	37.22 ± 3.15	0.904
NT-proBNP (pg/mL)	4815.92 ± 560.61	5601.26 ± 538.24	0.057	4934.39 ± 467.27	5906.79 ± 697.26	0.008	5204.51 ± 431.33	5316.10 ± 888.55	0.287
CTnI (pg/mL)	9435.84 ± 911.21	20,906.32 ± 1202.02	< 0.001	14,994.50 ± 975.91	16,642.27 ± 1426.50	0.094	15,550.19 ± 880.71	15,234.31 ± 2005.53	0.918
HDL-C (mmol/L)	1.30 ± 0.04	0.98 ± 0.02	< 0.001	1.24 ± 0.03	0.89 ± 0.02	< 0.001	1.19 ± 0.02	0.84 ± 0.02	< 0.001
LDL-C (mmol/L)	2.55 ± 0.06	2.66 ± 0.06	0.312	2.69 ± 0.05	2.43 ± 0.07	0.004	2.41 ± 0.04	3.62 ± 0.10	< 0.001
Total triglyceride (mmol/L)	1.36 ± 0.07	1.42 ± 0.07	0.113	1.33 ± 0.06	1.53 ± 0.10	0.012	1.36 ± 0.06	1.55 ± 0.09	0.002
Total cholesterol (mmol/L)	4.10 ± 0.07	4.14 ± 0.07	0.693	4.25 ± 0.06	3.84 ± 0.08	< 0.001	3.95 ± 0.05	4.98 ± 0.12	< 0.001
WBC count (10^9^/L)	6.72 ± 0.13	11.10 ± 0.21	< 0.001	8.30 ± 0.17	10.72 ± 0.31	< 0.001	8.97 ± 0.17	9.38 ± 0.38	0.780
Platelet count (10^9^/L)	191.70 ± 3.82	212.31 ± 4.01	< 0.001	200.59 ± 3.32	207.16 ± 5.27	0.379	200.23 ± 3.06	214.22 ± 7.08	0.372
Neutrophil count (10^9^/L)	4.56 ± 0.11	9.19 ± 0.21	< 0.001	6.36 ± 0.17	8.47 ± 0.33	< 0.001	6.98 ± 0.18	7.14 ± 0.37	0.357
Lymphocyte count (10^9^/L)	1.40 ± 0.04	1.37 ± 0.04	0.142	1.32 ± 0.03	1.53 ± 0.06	0.002	1.37 ± 0.03	1.47 ± 0.08	0.809
Monocyte count (10^9^/L)	0.45 ± 0.01	0.65 ± 0.02	< 0.001	0.43 ± 0.01	0.84 ± 0.02	< 0.001	0.55 ± 0.01	0.57 ± 0.03	0.145
Hemoglobin (mg/dL)	126.65 ± 1.19	125.87 ± 1.10	0.536	126.88 ± 0.95	124.78 ± 1.53	0.297	126.02 ± 0.86	127.34 ± 2.26	0.922
Medications in hospital, n (%)									
Antiplatelet therapy	243 (97.59%)	271 (97.13%)	0.744	361 (98.37%)	153 (95.03%)	0.051	427 (97.27%)	87 (97.75%)	0.003
Beta-blocker	182 (73.09%)	198 (70.97%)	0.587	271 (73.84%)	109 (67.70%)	< 0.001	317 (72.21%)	63 (70.79%)	0.237
ACEI/ARB	191 (76.71%)	187 (67.03%)	0.014	271 (73.84%)	107 (66.46%)	0.083	318 (72.44%)	60 (67.42%)	0.596
Statin	247 (99.20%)	275 (98.57%)	0.915	364 (99.18%)	158 (98.14%)	0.297	434 (98.86%)	88 (98.88%)	0.002
Long-term clinical events, n (%)									
Long-term mortality	17 (6.83%)	59 (21.15%)	< 0.001	40 (10.90%)	36 (22.36%)	0.001	50 (11.39%)	26 (29.21%)	< 0.001
Long-term RMI	10 (4.02%)	31 (11.11%)	0.002	22 (5.99%)	19 (11.80%)	0.022	29 (6.61%)	12 (13.48%)	0.010

*AMI* acute myocardial infarction, *NHR* neutrophil to high-density lipoprotein cholesterol ratio, *MHR* monocyte to high-density lipoprotein cholesterol ratio, *LDL-C/HDL-C* low-density lipoprotein cholesterol to high-density lipoprotein cholesterol ratio, *CAD* coronary artery disease, *CVD* cerebrovascular diseases, *SBP* systolic blood pressure, *DBP* diastolic blood pressure, *HR* heart rate, *STEMI* ST-segment elevation myocardial infarction, *NSTEMI* non-ST-elevation myocardial infarction, *LVEF* left ventricular ejection fraction, *AST* aspartate aminotransferase, *ALT* alanine aminotransferase, *NT-proBNP* N-terminal pro-brain natriuretic peptide, *CTnI* cardiac troponin I, *HDL-C* high-density lipoprotein cholesterol, *LDL-C* low-density lipoprotein cholesterol, *WBC* white blood cell, *ACEI/ARB* angiotensin-converting enzyme inhibitor/ angiotensin II receptor blocker, *RMI* recurrent myocardial infarction

Data presented are mean ± SEM or n(%)

In the groups divided by the NHR < 5.74 and NHR ≥ 5.74, results showed that there were clinically statistical differences among two groups in terms of medical history of diabetes (*P* = 0.007), vital signs at admission (*P* = 0.013; *P* < 0.001), LVEF (*P* = 0.001). Elevated level of NHR was associated with increased WBC and its subtypes (*P* < 0.001, respectively), so was in platelet count (*P* < 0.001), but not with lymphocyte count (*P* = 0.142) and hemoglobin (*P* = 0.536). Moreover, patients in NHR ≥ 5.74 were more likely to have worse hepatorenal function (*P* ≤ 0.001, respectively) and decreased concentration of HDL-C (*P* < 0.001).

In the groups divided by the MHR < 0.67 and MHR ≥ 0.67, it was noted that there were statistic differences in age (*P* = 0.048), gender (*P* < 0.001), smoking status (*P* = 0.021), the function index of heart and liver and kidney and the level of serum lipid as well as the complete blood counts (*P* < 0.050, respectively). In terms of the data grouped by the cut-off point of LDL-C/HDL-C, no statistically significant between-group differences for the primary outcomes were detected, except for the level of serum lipids (*P* < 0.001, *P* < 0.001, *P* < 0.001, *P* = 0.002, respectively).

The angiographic characteristics were reported in Table 2. When grouping by NHR, no significant differences were discovered in the culprit vessel location and the CABG therapy, except for the culprit vessel quantity (*P* < 0.001), onset to reperfusion time (*P* < 0.001), tirofiban use (*P* = 0.038), stent use (*P* = 0.030), thrombus aspiration use (*P* = 0.001), the multi-vessel PCI therapy (*P* = 0.006), the TIMI grade (*P* < 0.001) and Gensini score (*P* = 0.003). In the groups divided by the MHR < 0.67 and MHR ≥ 0.67, it was resulted that the CAG outcomes were worse in the high-risk group. What’s more, it was the same in the LDL-C/HDL-C grouping.

**Table 2:** Angiographic and procedural characteristics in the study population

Variable	NHR < 5.74	NHR ≥ 5.74	*P*	MHR < 0.67	MHR ≥ 0.67	*P*	LDL-C/HDL-C < 3.57	LDL-C/HDL-C ≥ 3.57	*P*
	*n* = 249	*n* = 279		*n* = 367	*n* = 161		*n* = 439	*n* = 89	
Culprit vessel, n (%)									
LAD	175 (70.28%)	216 (77.42%)	0.062	273 (74.39%)	118 (73.29%)	< 0.001	325 (74.03%)	66 (74.16%)	0.980
LCX	128 (51.41%)	160 (57.35%)	0.171	193 (52.59%)	95 (59.01%)	0.173	234 (53.30%)	54 (60.67%)	0.203
RCA	130 (52.21%)	167 (59.86%)	0.077	209 (56.95%)	88 (54.66%)	0.625	245 (55.81%)	52 (58.43%)	0.650
No. of diseased vessels, n (%)			< 0.001			< 0.001			< 0.001
1	55 (22.09%)	67 (24.01%)		95 (25.89%)	27 (16.77%)		105 (23.92%)	17 (19.10%)	
2	60 (24.10%)	70 (25.09%)		86 (23.43%)	44 (27.33%)		105 (23.92%)	25 (28.09%)	
3	87 (34.94%)	112 (40.14%)		137 (37.33%)	62 (38.51%)		163 (37.13%)	35 (39.33%)	
Onset to reperfusion time, h	16.43 ± 0.56	12.95 ± 0.54	< 0.001	14.66 ± 0.46	14.45 ± 0.75	0.698	14.67 ± 0.43	14.24 ± 0.98	0.443
Tirofiban use, n (%)	170 (68.27%)	213 (76.34%)	0.038	276 (75.20%)	107 (66.46%)	0.038	321 (73.12%)	62 (69.66%)	0.505
Stent use, n (%)	146 (58.63%)	189 (67.74%)	0.030	242 (65.94%)	93 (57.76%)	0.072	279 (63.55%)	55 (61.80%)	0.754
Use of thrombus aspiration, n (%)	8 (3.21%)	29 (10.39%)	0.001	30 (8.17%)	8 (4.97%)	0.189	30 (6.83%)	7 (7.87%)	0.728
Multi-vessel PCI therapy, n (%)	201 (80.72%)	249 (89.25%)	0.006	317 (86.38%)	133 (82.61%)	0.261	373 (84.97%)	77 (86.52%)	0.707
CABG therapy, n (%)	21 (8.43%)	17 (6.09%)	0.299	25 (6.81%)	14 (8.70%)	0.446	29 (6.61%)	9 (10.11%)	0.243
Preprocedural TIMI grade, n (%):			< 0.001			< 0.001			< 0.001
0	118 (47.39%)	181 (64.87%)		206 (56.13%)	93 (57.76%)		242 (55.13%)	58 (65.17%)	
1	73 (29.32%)	64 (22.94%)		101 (27.52%)	36 (22.36%)		119 (27.11%)	18 (20.22%)	
2	9 (3.61%)	3 (1.08%)		10 (2.72%)	3 (1.86%)		11 (2.51%)	1 (1.12%)	
3	0 (0.00%)	1 (0.36%)		0 (0.00%)	1 (0.62%)		1 (0.23%)	0 (0.00%)	
Postprocedural TIMI grade, n (%):			< 0.001			< 0.001			< 0.001
0	31 (12.45%)	34 (12.19%)		93 (25.34%)	50 (31.06%)		50 (11.39%)	16 (17.98%)	
1	14 (5.62%)	18 (6.45%)		22 (5.99%)	10 (6.21%)		27 (6.15%)	5 (5.62%)	
2	8 (3.21%)	7 (2.51%)		12 (3.27%)	3 (1.86%)		15 (3.42%)	0 (0.00%)	
3	148 (59.44%)	189 (67.74%)		240 (65.40%)	98 (60.87%)		281 (64.01%)	56 (62.92%)	
Gensini score	70.92 ± 3.61	84.78 ± 3.35	0.003	78.60 ± 3.06	77.43 ± 4.13	0.662	75.45 ± 2.66	92.03 ± 6.34	0.005

*NHR* neutrophil to high-density lipoprotein cholesterol ratio, *MHR* monocyte to high-density lipoprotein cholesterol ratio, *LDL-C/HDL-C* low-density lipoprotein cholesterol to high-density lipoprotein cholesterol ratio, *LAD* left coronary artery, *LCX* left circumflex, *RCA* right coronary artery, *PCI* percutaneous coronary intervention, *CABG* coronary artery bypass grafting, *TIMI* thrombolysis in myocardial infarction

Data presented are mean ± SEM or n(%)

### Survival analysis

**Fig. 2:**
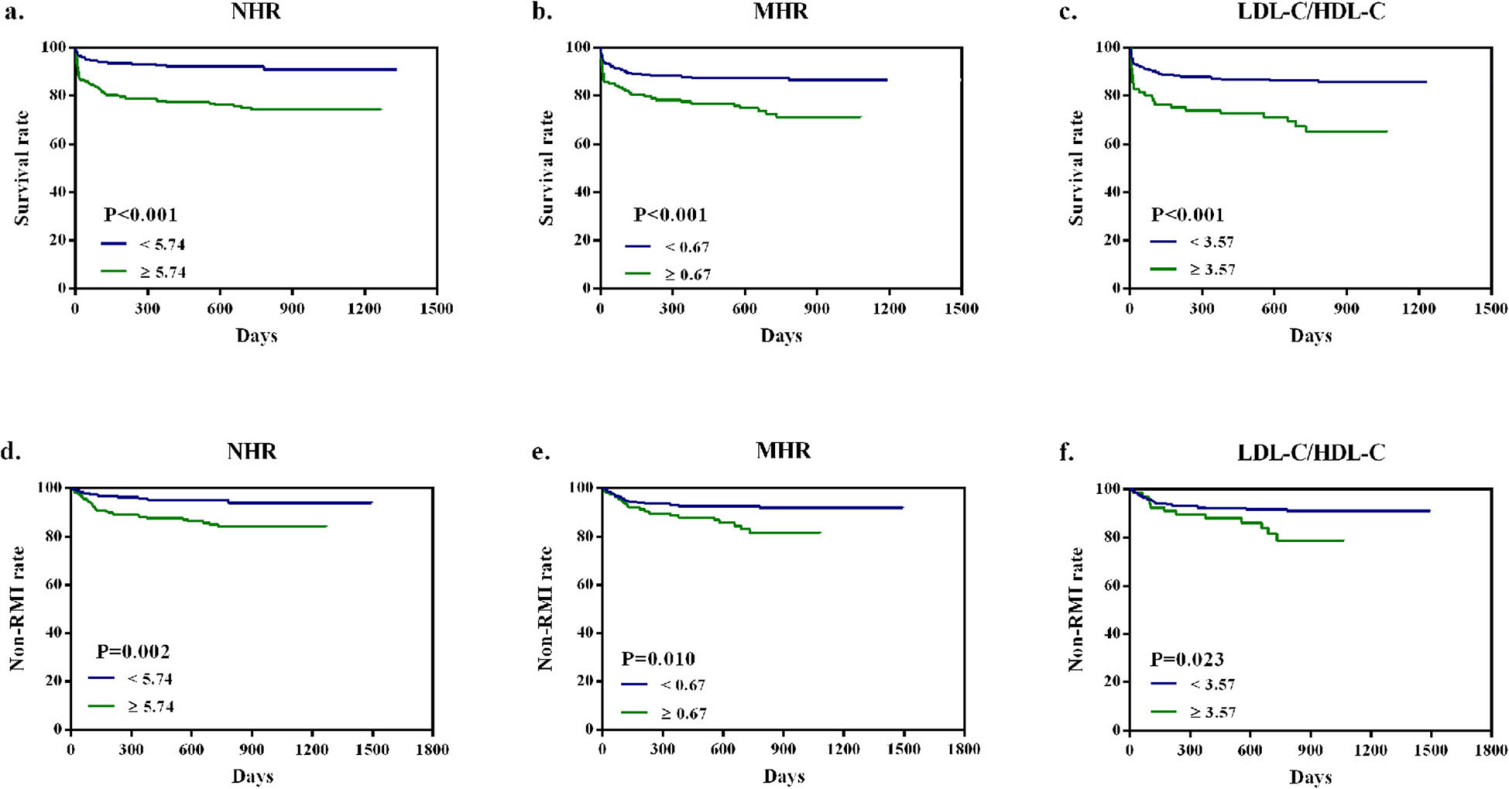
Kaplan-Meier survival curves for long-term mortality and RMI according to the NHR, MHR and LDL-C/HDL-C (**a, b, c, d, e, f**). **a** Kaplan-Meier survival curves of long-term mortality according to NHR (log-rank test: *P* < 0.001). **b** Kaplan-Meier survival curves of long-term mortality according to MHR (log-rank test: *P* < 0.001). **c** Kaplan-Meier survival curves of long-term mortality according to LDL-C/HDL-C (log-rank test: *P* < 0.001). **d** Kaplan-Meier survival curves of long-term RMI according to NHR (log-rank test: *P* = 0.002). **e** Kaplan-Meier survival curves of long-term RMI according to MHR (log-rank test: *P* = 0.010). **f** Kaplan-Meier survival curves of long-term RMI according to LDL-C/HDL-C (log-rank test: *P* = 0.023). RMI, recurrent myocardial infarction. NHR, neutrophil to high-density lipoprotein cholesterol ratio. MHR, monocyte to high-density lipoprotein cholesterol ratio. LDL-C/HDL-C, low-density lipoprotein cholesterol to high-density lipoprotein cholesterol ratio

The Kaplan-Meier Curve were plotted with the event-free surival data from the follow-up. Mean duration of follow-up was 673.85 ± 14.32 days (median 679.50 days). The long-term mortality in the high-risk groups were significantly higher than low-risk groups (log-rank tests: all *P* < 0.001, Fig. 2a-c). As shown in Fig. 2d-f, it was indicated that patients in the high-risk groups had significantly worse long-term RMI than patients in the low-risk groups (log-rank tests: *P* = 0.002, *P* = 0.010, *P* = 0.023, respectively).

### Univariate and multivariate cox regression analyses

In multivariate regression analysis, Gensini score (hazard ratio [HR]: 1.00, 95% CI: 1.00 to 1.01, *P* = 0.034), Killip class (HR: 1.85, 95% CI: 1.48 to 2.31, *P* < 0.001), WBC (HR:1.09, 95% CI: 1.03 to 1.16, *P* = 0.005), hemoglobin (HR: 0.98, 95% CI: 0.97 to 0.99, *P* = 0.001) and the NHR (HR: 1.96, 95% CI: 1.02 to 3.75, *P* = 0.044) were found as independent predictors of long-term mortality, but not with MHR (*P* = 0.237) or LDL-C/HDL-C (*P* = 0.092) (Table 3). Moreover, for predicting long-term RMI, we found that NHR (HR: 2.23, 95% CI: 1.04 to 4.79, *P* = 0.040) was still the potential predictor along with gender (HR: 2.27, 95% CI: 1.22 to 4.23, *P* = 0.010), Killip class (HR: 1.69, 95% CI: 1.23 to 2.33, *P* = 0.001) and monocyte (HR: 3.47, 95% CI: 1.46 to 8.26, *P* = 0.005), but not with MHR (*P* = 0.505) and LDL-C/HDL-C (*P* = 0.248) (Table 4).

**Table 3:** Predictors of long-term mortality in univariable and multivariable Cox regression analyses

Variables	Univariable		Multivariable	
	Hazard ratio (95% CI)	*P*	Hazard ratio (95% CI)	*P*
Age, year	1.09 (1.04 to 1.13)	< 0.001	–	0.268
Gender (male)	1.73 (1.10 to 2.71)	0.017	–	0.066
Gensini score	1.01 (1.00 to 1.01)	0.009	**1.00 (1.00 to 1.01)**	**0.034**
Smoking status	1.53 (0.94 to 2.50)	0.087	–	0.814
Hypertension	1.09 0.69 to 1.73)	0.703	–	0.877
Prior CAD	1.47 (0.82 to 2.62)	0.196	–	0.245
Diabetes	1.85 (1.17 to 2.94)	0.009	–	0.225
Killip class	2.14 (1.73 to 2.64)	< 0.001	**1.85 (1.48 to 2.31)**	**< 0.001**
LVEF	0.97 (0.95 to 0.98)	< 0.001	–	0.274
CTnI	1.97 (1.26 to 3.09)	0.003	–	0.333
WBC	1.16 (1.11 to 1.23)	< 0.001	**1.09 (1.03 to 1.16)**	**0.005**
Neutrophil	1.17 (1.11 to 1.22)	< 0.001	–	0.866
Lymphocyte	0.89 (0.62 to 1.26)	0.499		
Monocyte	2.84 (1.50 to 5.37)	0.001	–	0.496
Platelet	1.00 (1.00 to 1.01)	0.102		
Hemoglobin	0.98 (0.97 to 0.99)	< 0.001	**0.98 (0.97 to 0.99)**	**0.001**
Total cholesterol	1.12 (0.93 to 1.35)	0.246		
Total triglyceride	0.95 (0.76 to 1.18)	0.628		
HDL-C	0.64 (0.35 to 1.16)	0.139		
LDL-C	1.18 (0.95 to 1.48)	0.130		
Creatinine	1.01 (1.01 to 1.02)	< 0.001	–	0.207
NHR ≥ 5.74	3.21 (1.87 to 5.50)	< 0.001	**1.96 (1.02 to 3.75)**	**0.044**
MHR ≥ 0.67	2.22 (1.42 to 3.49)	0.001	–	0.237
LDL-C/HDL-C ≥ 3.57	1.25 (1.59 to 4.11)	0.010	–	0.092

*CI* confidence interval, *CAD* coronary artery disease, *LVEF* left ventricular ejection fraction, *CTnI* cardiac troponin I, *WBC* white blood cell, *HDL-C* high-density lipoprotein cholesterol, *LDL-C* low-density lipoprotein cholesterol, *NHR* neutrophil to high-density lipoprotein cholesterol ratio, *MHR* monocyte to high-density lipoprotein, *LDL-C/HDL-C* low-density lipoprotein cholesterol to high-density lipoprotein cholesterol ratio

Note: Bolded differences show statistical difference at the *p* < 0.05 for multivariate analysis

**Table 4:** Predictors of long-term RMI in univariable and multivariable Cox regression analyses

Variables	Univariable		Multivariable	
	Hazard ratio (95% CI)	*P*	Hazard ratio (95% CI)	*P*
Age, year	1.06 (1.01 to 1.13)	0.028	–	0.275
Gender (male)	2.15 (1.16 to 3.92)	0.015	**2.27 (1.22 to 4.23)**	**0.010**
Gensini score	1.01 (1.00 to 1.01)	0.046	–	0.104
Smoking status	1.46 (0.75 to 2.81)	0.263	–	0.753
Hypertension	1.00 (0.54 to 1.87)	0.990	–	0.992
Prior CAD	1.95 (0.60 to 6.33)	0.265	–	0.401
Diabetes	2.18 (1.17 to 4.05)	0.014	–	0.240
Killip class	1.73 (1.28 to 2.34)	< 0.001	**1.69 (1.23 to 2.33)**	**0.001**
LVEF	0.97 (0.95 to 1.00)	0.026	–	0.837
CTnI	1.86 (1.01 to 3.43)	0.048	–	0.236
WBC	1.15 (1.07 to 1.24)	< 0.001	–	0.554
Neutrophil	1.14 (1.07 to 1.23)	< 0.001	–	0.797
Lymphocyte	0.78 (0.47 to 1.30)	0.342		
Monocyte	4.31 (1.93 to 9.58)	< 0.001	**3.47 (1.46 to 8.26)**	**0.005**
Platelet	1.01 (1.00 to 1.01)	0.009	–	0.075
Hemoglobin	0.98 (0.97 to 1.00)	0.012	–	0.089
Total cholesterol	1.21 (0.95 to 1.56)	0.126		
Total triglyceride	1.02 (0.79 to 1.31)	0.906		
HDL-C	0.94 (0.49 to 1.81)	0.845		
LDL-C	1.25 (0.94 to 1.67)	0.122		
Creatinine	1.01 (1.00 to 1.02)	0.049	–	0.276
NHR ≥ 5.74	2.98 (1.46 to 6.08)	0.003	**2.23 (1.04 to 4.79)**	**0.040**
MHR ≥ 0.67	2.20 (1.19 to 4.07)	0.012	–	0.505
LDL-C/HDL-C ≥ 3.57	2.14 (1.09 to 4.20)	0.027	–	0.248

*RMI* recurrent myocardial infarction, *CI* confidence interval, *CAD* coronary artery disease, *LVEF* left ventricular ejection fraction, *CTnI* cardiac troponin I, *WBC* white blood cell, *HDL-C* high-density lipoprotein cholesterol, *LDL-C* low-density lipoprotein cholesterol, *NHR* neutrophil to high-density lipoprotein cholesterol ratio, *MHR* monocyte to high-density lipoprotein cholesterol ratio, *LDL-C/HDL-C* low-density lipoprotein cholesterol to high-density lipoprotein cholesterol ratio

Note: Bolded differences show statistical difference at the p < 0.05 for multivariate analysis

### Correlation analysis between lipid ratios and Gensini score

**Fig. 3:**
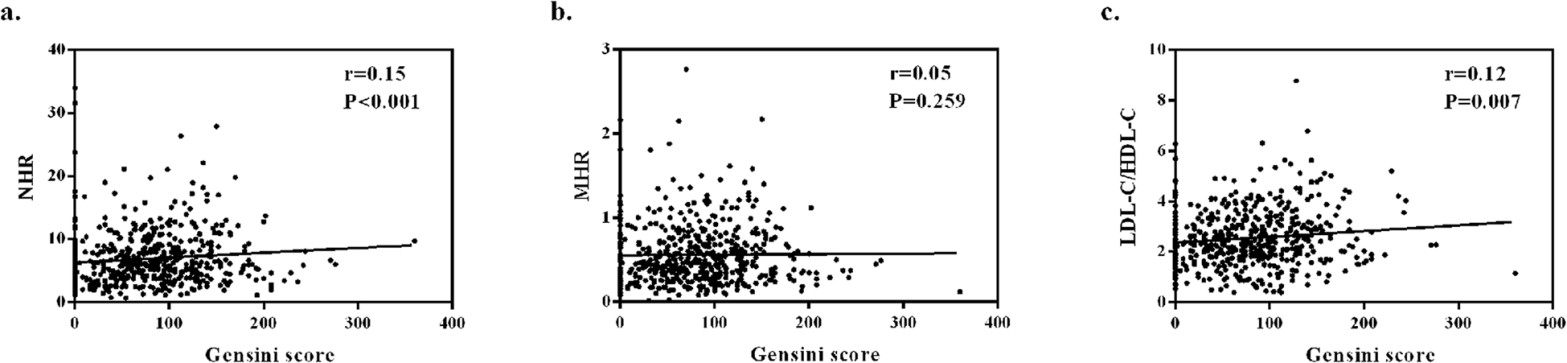
Correlation between lipid ratios (NHR, MHR and LDL-C/HDL-C) and Gensini score in AMI patients (**a, b, c**). **a.** There was a positive correlation between NHR and Gensini score: *r* = 0.15, *P* < 0.001. **b.** There was no correlation between MHR and Gensini score: *r* = 0.05, *P* = 0.259. **c.** There was a positive correlation between LDL-C/HDL-C and Gensini score: *r* = 0.12, *P* = 0.007. AMI, acute myocardial infarction. NHR, neutrophil to high-density lipoprotein cholesterol ratio. MHR, monocyte to high-density lipoprotein cholesterol ratio. LDL-C/HDL-C, low-density lipoprotein cholesterol to high-density lipoprotein cholesterol ratio

As shown in Fig. 3, a weak but significant positive correlation between NHR and Gensini score in our population (*r* = 0.15, *P* < 0.001; Fig. 3a), so was in LDL-C/HDL-C (*r* = 0.12, *P* = 0.007; Fig. 3c). It was found that there was no correlation between MHR and Gensini score (*r* = 0.05, *P* = 0.259; Fig. 3b).

## Discussion

In our work, our results presented that higher level of NHR was associated with higher risk of long-term mortality and RMI. NHR has a superior prognostic value for long-term clinical outcomes in elderly patients compared with MHR and LDL-C/HDL-C. Moreover, there was a positive correlation between NHR and the severity of a coronary artery. This study appears to be novel to assess the prognostic role of NHR for long-term outcomes in elderly AMI patients. The results of present study mainly applied to patients aged between 65 and 85 years.

Recent studies have payed more attentions to the linkage between lipid-related biomarkers and cardiac disease. Previous studies reported that the elevated MHR has greater risk of the worse prognosis in AMI [25–27]. Besides, in a prospective cohort study, the high level of LDL-C/HDL-C was discovered to be related with an increased risk of adverse cardiovascular events [28]. From the survival analysis, our results were consistent with previous studies. Actually, the etiology of AMI was complicated, included alternated lipid metabolism, inflammatory reaction, oxidation injury, and other pathological processes [29–32]. Herein, we hypothesized that the NHR, a novel index composed of inflammatory cell and lipid cholesterol, might reflect the inflammatory status and the lipid metabolism more comprehensively. In the present study, the prediction abilities of NHR and other two existing ratios (MHR and LDL-C/HDL-C) were compared in univariate and multivariate analyses. After adjusting for confounding factors, our results suggested a possible better prediction ability for NHR levels in long-term clinical outcomes, compared with MHR and LDL-C/HDL-C.

From our analysis, we attempted to explain the result that higher NHR was associated with poorer clinical outcomes. Neutrophil was an indispensable component participating in the immuno-inflammatory response of AMI [33, 34]. An increase in neutrophil has been verified at the site of coronary lesion [35]. Recent evidences indicated that elevated level of myeloperoxidase from the neutrophil could give rise to coronary atherosclerosis [36, 37]. Yunoki et al. pointed out that plasma myeloperoxidase levels were inversely correlated with HDL-C and serum paraoxonase-1 (PON-1) concentrations and activities [38]. It was supposed that the interaction between myeloperoxidase and HDL-C in the circulation may decrease serum PON-1 levels. Besides, Park et al. reported that the risk of cardiovascular death and RMI appeared higher in the patients with lower level of HDL-C level [39]. Previous studies showed that HDL-C was engaged in the inhibition of the inflammation and oxidation [40, 41]. According to the Murphy’s study, the ability of HDL-C to inhibit neutrophils activation, attachment, diffusion, and migration might be related to the abundance of lipid rafts [16].

Above all, because of the interaction of increased neutrophils and decreased HDL-C in AMI, an elevated NHR might be related to the inflammatory activity and the abnormal lipids metabolism. Besides, as for the complex interactions between neutrophil and HDL-C, the combined indicator of NHR might be more reliable than single parameter. That was consistent with the results from the present study. On top of all, as a new predictor for the prognosis of AMI patient, NHR could be calculated from the complete blood count on admission, which is quick and convenient.

Gensini score was an indicator to evaluate the severity of coronary artery disease [42]. Specifically, different weight coefficients were assigned to different coronary artery branches according to their severity, which was conducive to reflect the severity of the disease objectively. Based on our results, NHR was positively correlated with Gensini score and was an effective predictive factor for long-term clinical outcomes. The combined application of the NHR and Gensini score might be more helpful for clinicians to identify the high-risk patients with AMI. Further studies are needed to validate this hypothesis.

We have to acknowledge that there were some limitations in this study. First, the retrospective design of the study sets a limit to the convincement of our study. Secondly, we could not compare the lipid ratios with other conventional inflammatory markers, for instance, C-reactive protein, fibrinogen, or myeloperoxidase. Because they were not routinely obtained in our study population. Thirdly, the patients were admitted with AMI within one month of symptom onset. Thus, the neutrophil level might not be static across this period of time. Lastly, our results did not show the well established link between diabetes and mortality. It might be because of the differences in sample size, study design, population selection, statistical method, outcome measurement and so on.

## Conclusion

In summary, we observed that NHR might has a predictive value for prognosis in the long-term mortality and RMI of elderly AMI patients, which is superior to both MHR and LDL-C/HDL-C.

## Data Availability

The data analyzed in the current study are available from the corresponding authors on reasonable request.

## Abbreviations

AMI: Acute myocardial infarction
HDL-C: High-density lipoprotein cholesterol
NHR: Neutrophil to high-density lipoprotein cholesterol ratio
MHR: Monocyte to high-density lipoprotein cholesterol ratio
LDL-C: Low-density lipoprotein cholesterol
LDL-C/HDL-C: Low-density lipoprotein cholesterol to high-density lipoprotein cholesterol ratio
ROC: Receiver operating characteristic
RMI: Recurrent myocardial infarction
CAD: Coronary artery disease
STEMI: ST-segment elevation myocardial infarction
NSTEMI: Non-ST-elevation myocardial infarction
WBC: White blood cell
LVEF: Left ventricular ejection fraction
CAG: Coronary angiography
PCI: Percutaneous coronary intervention
CABG: Coronary artery bypass grafting
TIMI: Thrombolysis in myocardial infarction
AUC: Area under the curve
CI: Confidence interval
HR: Hazard ratio
PON-1: Paraoxonase-1

## References

[1] Arora S, Stouffer GA, Kucharska-Newton AM, Qamar A, Vaduganathan M, Pandey A (2019). Twenty year trends and sex differences in young adults hospitalized with acute myocardial infarction. Circulation..

[2] Xiao J, Xu F, Yang CL, Chen WQ, Chen X, Zhang H (2018). Preferred revascularization strategies in patients with ischemic heart failure: a meta-analysis. Curr Med Sci.

[3] Body R, Carley S, McDowell G, Jaffe AS, France M, Cruickshank K (2011). Rapid exclusion of acute myocardial infarction in patients with undetectable troponin using a high-sensitivity assay. J Am Coll Cardiol.

[4] Winter MP, Wiesbauer F, Blessberger H, Pavo N, Sulzgruber P, Huber K (2018). Lipid profile and long-term outcome in premature myocardial infarction. Eur J Clin Investig.

[5] Karthikeyan G, Teo KK, Islam S, McQueen MJ, Pais P, Wang X (2009). Lipid profile, plasma apolipoproteins, and risk of a first myocardial infarction among Asians: an analysis from the INTERHEART study. J Am Coll Cardiol.

[6] Prospective Studies C, Lewington S, Whitlock G, Clarke R, Sherliker P, Emberson J (2007). Blood cholesterol and vascular mortality by age, sex, and blood pressure: a meta-analysis of individual data from 61 prospective studies with 55,000 vascular deaths. Lancet..

[7] Canouï-Poitrine F, Luc G, Bard JM, Ferrieres J, Yarnell J, Arveiler D (2010). Relative contribution of lipids and Apolipoproteins to incident coronary heart disease and ischemic stroke: the PRIME study. Cerebrovasc Dis.

[8] Ganjali S, Gotto AM, Ruscica M, Atkin SL (2018). Monocyte-to-HDL-cholesterol ratio as a prognostic marker in cardiovascular diseases. J Cell Physiol.

[9] Zhang Y, Li S, Guo YL, Wu NQ, Zhu CG, Gao Y (2016). Is monocyte to HDL ratio superior to monocyte count in predicting the cardiovascular outcomes: evidence from a large cohort of Chinese patients undergoing coronary angiography. Ann Med.

[10] Kastelein JJ, van der Steeg WA, Holme I, Gaffney M, Cater NB, Barter P (2008). Lipids, apolipoproteins, and their ratios in relation to cardiovascular events with statin treatment. Circulation..

[11] Zhong Z, Hou J, Zhang Q, Zhong W, Li B, Li C (2019). Assessment of the LDL-C/HDL-C ratio as a predictor of one year clinical outcomes in patients with acute coronary syndromes after percutaneous coronary intervention and drug-eluting stent implantation. Lipids Health Dis.

[12] Hansson GK (2005). Inflammation, atherosclerosis, and coronary artery disease. N Engl J Med.

[13] Bartels ED, Christoffersen C, Lindholm MW, Nielsen LB (2015). Altered metabolism of LDL in the arterial wall precedes atherosclerosis regression. Circ Res.

[14] Forrester JS (2002). Prevention of plaque rupture: a new paradigm of therapy. Ann Intern Med.

[15] Guo TM, Cheng B, Li KE, Guan SM, Qi BL, Li WZ (2018). Prognostic value of neutrophil to lymphocyte ratio for in-hospital mortality in elderly patients with acute myocardial infarction. Curr Med Sci..

[16] Murphy AJ, Woollard KJ, Suhartoyo A, Stirzaker RA, Shaw J, Sviridov D (2011). Neutrophil activation is attenuated by high-density lipoprotein and Apolipoprotein A-I in in vitro and in vivo models of inflammation. Arterioscler Thromb Vasc Biol.

[17] Soehnlein O (2009). An elegant defense: how neutrophils shape the immune response. Trends Immunol.

[18] Wang HH, Garruti G, Liu M, Portincasa P, Wang Q-HD (2017). Cholesterol and lipoprotein metabolism and atherosclerosis: recent advances in reverse cholesterol transport. Ann Hepatol.

[19] Curcic S, Holzer M, Frei R, Pasterk L, Schicho R, Heinemann A (1851). Neutrophil effector responses are suppressed by secretory phospholipase A2 modified HDL. Biochim Biophys Acta.

[20] Cogny A, ATGER, Véronique PJL, Soni T, Moatti N (1996). High-density lipoprotein 3 physicochemical modifications induced by interaction with human polymorphonuclear leucocytes affect their ability to remove cholesterol from cells. Biochem J.

[21] O'Gara PT, Kushner FG, Ascheim DD, Casey DE, Chung MK, Lemos JA (2013). 2013 ACCF/AHA guideline for the management of ST-elevation myocardial infarction: a report of the American College of Cardiology Foundation/American Heart Association task force on practice guidelines. Circulation..

[22] Amsterdam EA, Wenger NK, Brindis RG, Casey DE, Ganiats TG, Holmes DR (2014). 2014 AHA/ACC guideline for the Management of Patients with non-ST-elevation acute coronary syndromes: a report of the American College of Cardiology/American Heart Association task force on practice guidelines. J Am Coll Cardiol.

[23] Windecker S, Kolh P, Alfonso F, Collet JP, Cremer J, Falk V (2014). 2014 ESC/EACTS guidelines on myocardial revascularization: the task force on myocardial revascularization of the European Society of Cardiology (ESC) and the European Association for Cardio-Thoracic Surgery (EACTS) developed with the special contribution of the European Association of Percutaneous Cardiovascular Interventions (EAPCI). Eur Heart J.

[24] Nakatani D, Sakata Y, Suna S, Usami M, Matsumoto S, Shimizu M (2013). Incidence, predictors, and subsequent mortality risk of recurrent myocardial infarction in patients following discharge for acute myocardial infarction. Circ J.

[25] Canpolat U, Cetin EH, Cetin S, Aydin S, Akboga MK, Yayla C (2016). Association of Monocyte-to-HDL cholesterol ratio with slow coronary flow is linked to systemic inflammation. Clin Appl Thromb Hemost.

[26] Kundi H, Gok M, Kiziltunc E, Cetin M, Cicekcioglu H, Cetin ZG (2015). Relation between monocyte to high-density lipoprotein cholesterol ratio with presence and severity of isolated coronary artery Ectasia. Am J Cardiol.

[27] Cetin EH, Cetin MS, Canpolat U, Aydin S, Topaloglu S, Aras D (2015). Monocyte/HDL-cholesterol ratio predicts the definite stent thrombosis after primary percutaneous coronary intervention for ST-segment elevation myocardial infarction. Biomark Med.

[28] Knunutsor SK, Francesco Z, Jouni K, Kurl S, Laukkanen JA (2017). Is high serum LDL/HDL cholesterol ratio an emerging risk factor for sudden cardiac death? Findings from the KIHD study. J Atheroscler Thromb.

[29] Wilson PW (2008). Evidence of systemic inflammation and estimation of coronary artery disease risk: a population perspective. Am J Med.

[30] Kehl DW, Iqbal N, Fard A, Kipper BA, De La Parra LA, Maisel AS (2012). Biomarkers in acute myocardial injury. Transl Res.

[31] Hickman PE, McGill DA, Talaulikar G, Hiremagalur B, Bromley J, Rahman A (2009). Prognostic efficacy of cardiac biomarkers for mortality in dialysis patients. Intern Med J.

[32] Xu LW, Su YY, Zhao YC, Sheng XC, Tong RY, Ying XY (2019). Melatonin differentially regulates pathological and physiological cardiac hypertrophy: Crucial role of circadian nuclear receptor RORα signaling. J Pineal Res.

[33] Montecucco F, Liberale L, Bonaventura A, Vecchie A, Dallegri F, Carbone F (2017). The role of inflammation in cardiovascular outcome. Curr Atheroscler Rep.

[34] Ding S, Nan L, Sheng XC, Zhao YC, Su YY, Xu LW (2019). Melatonin stabilizes rupture-prone vulnerable plaques via regulating macrophage polarization in a nuclear circadian receptor RORα-dependent manner. J Pineal Res.

[35] Pierre R, Thams S, Soehnlein O, Kenne E, Tseng CN, Björkström NK (2010). Distinct infiltration of neutrophils in lesion shoulders in ApoE−/− mice. Am J Pathol.

[36] Goldmann BU, Rudolph V, Rudolph TK, Holle AK, Hillebrandt M, Meinertz T (2009). Neutrophil activation precedes myocardial injury in patients with acute myocardial infarction. Free Radic Biol Med.

[37] Wang XS, Kim HB, Szuchman-Sapir A, McMahon A, Dennis JM, Witting PK (2016). Neutrophils recruited to the myocardium after acute experimental myocardial infarct generate hypochlorous acid that oxidizes cardiac myoglobin. Arch Biochem Biophys.

[38] Yunoki K, Naruko T, Inaba M, Inoue T, Nakagawa M, Sugioka K (2013). Gender-specific correlation between plasma myeloperoxidase levels and serum high-density lipoprotein-associated paraoxonase-1 levels in patients with stable and unstable coronary artery disease. Atherosclerosis..

[39] Park JS, Cha KS, Lee HW, Oh JH, Choi JH, Lee HC (2018). Predictive and protective role of high-density lipoprotein cholesterol in acute myocardial infarction. Cardiol J.

[40] Gomaraschi M, Calabresi L, Franceschini G (2016). Protective effects of HDL against ischemia/reperfusion injury. Front Pharmacol.

[41] Murphy AJ, Woollard KJ, Hoang A, Mukhamedova N, Stirzaker RA, McCormick SP (2008). High-density lipoprotein reduces the human monocyte inflammatory response. Arterioscler Thromb Vasc Biol.

[42] Iscanli MD, Metin Aksu N, Evranos B, Aytemir K, Ozmen MM (2014). Comparison of TIMI and Gensini score in patients admitted to the emergency department with chest pain, who underwent coronary angiography. Med Sci Monit.

